# Estimates of genomic inbreeding and identification of candidate regions that differ between Chinese indigenous sheep breeds

**DOI:** 10.1186/s40104-021-00608-9

**Published:** 2021-08-05

**Authors:** Jiaxin Liu, Liangyu Shi, Yang Li, Liang Chen, Dorian Garrick, Lixian Wang, Fuping Zhao

**Affiliations:** 1 Key Laboratory of Animal Genetics, Breeding and Reproduction (Poultry) of Ministry of Agriculture, Institute of Animal Sciences, Chinese Academy of Agricultural Sciences, Beijing, 100193 China; 2 The Affiliated High School of Peking University, Beijing, 100192 China; 3 A.L. Rae Centre of Genetics and Breeding, Massey University, Hamilton, 3240 New Zealand

**Keywords:** Candidate genes, Genomic inbreeding coefficient, ROH islands, Runs of homozygosity, Sheep

## Abstract

**Background:**

A run of homozygosity (ROH) is a consecutive tract of homozygous genotypes in an individual that indicates it has inherited the same ancestral haplotype from both parents. Genomic inbreeding can be quantified based on ROH. Genomic regions enriched with ROH may be indicative of selection sweeps and are known as ROH islands. We carried out ROH analyses in five Chinese indigenous sheep breeds; Altay sheep (*n* = 50 individuals), Large-tailed Han sheep (*n* = 50), Hulun Buir sheep (*n* = 150), Short-tailed grassland sheep (*n* = 150), and Tibetan sheep (*n* = 50), using genotypes from an Ovine Infinium HD SNP BeadChip.

**Results:**

A total of 18,288 ROH were identified. The average number of ROH per individual across the five sheep breeds ranged from 39 (Hulun Buir sheep) to 78 (Large-tailed Han sheep) and the average length of ROH ranged from 0.929 Mb (Hulun Buir sheep) to 2.544 Mb (Large-tailed Han sheep). The effective population size (*Ne*) of Altay sheep, Large-tailed Han sheep, Hulun Buir sheep, Short-tailed grassland sheep and Tibetan sheep were estimated to be 81, 78, 253, 238 and 70 five generations ago. The highest ROH-based inbreeding estimate (*F*_ROH_) was 0.0808 in Large-tailed Han sheep, whereas the lowest *F*_ROH_ was 0.0148 in Hulun Buir sheep. Furthermore, the highest proportion of long ROH fragments (> 5 Mb) was observed in the Large-tailed Han sheep breed which indicated recent inbreeding. In total, 49 ROH islands (the top 0.1% of the SNPs most commonly observed in ROH) were identified in the five sheep breeds. Three ROH islands were common to all the five sheep breeds, and were located on OAR2: 12.2–12.3 Mb, OAR12: 78.4–79.1 Mb and OAR13: 53.0–53.6 Mb. Three breed-specific ROH islands were observed in Altay sheep (OAR15: 3.4–3.8 Mb), Large-tailed Han sheep (ORA17: 53.5–53.8 Mb) and Tibetan sheep (ORA5:19.8–20.2 Mb). Collectively, the ROH islands harbored 78 unique genes, including 19 genes that have been documented as having associations with tail types, adaptation, growth, body size, reproduction or immune response.

**Conclusion:**

Different ROH patterns were observed in five Chinese indigenous sheep breeds, which reflected their different population histories. Large-tailed Han sheep had the highest genomic inbreeding coefficients and the highest proportion of long ROH fragments indicating recent inbreeding. Candidate genes in ROH islands could be used to illustrate the genetic characteristics of these five sheep breeds. Our findings contribute to the understanding of genetic diversity and population demography, and help design and implement breeding and conservation strategies for Chinese sheep.

**Supplementary Information:**

The online version contains supplementary material available at 10.1186/s40104-021-00608-9.

## Introduction

Selection is one of the main forces reshaping the genomes of domestic animals. A genomic region subjected to intense selection would leave a footprint as a result of the selection and this is known as a selection signature. These signatures might demonstrate increased frequency of favorable allele(s), and reduced nucleotide diversity around the selected locus [1]. The reduction in genetic variation can be characterized as consecutive segments of homozygous genotypes (i.e. runs of homozygosity; ROH). In animal breeding, selection plays the vital role in achieving genetic gain. Mating among related animals cannot be avoided because only a small proportion of individuals are elite, and these tend to be used more widely than average animals. Inbreeding results in loss of genetic diversity, the emergence of harmful recessive mutations, as well as reducing productive performance, notably fecundity. All these effects impact the profitability and sustainability of livestock and poultry [2, 3].

Traditionally, inbreeding was characterized in terms of the inbreeding coefficient, calculated according to pedigree records (*F*_PED_) as proposed by Wright [4]. *F*_PED_ reflects the probability that a pair of alleles is identical by descent (IBD), which is a statistical expectation [5]. Moreover, *F*_PED_ is usually underestimated because it assumes the founder animals in the pedigree are unrelated in which case *F*_PED_ ignores historical inbreeding prior to the founders [6–8]. The availability of genome sequencing data and high-density SNP genotypic data provides an opportunity to evaluate inbreeding at a molecular level. Inbreeding evaluation based on genomic information could reflect the true inbreeding value by directly identifying alleles at a locus that are IBD.

An individual inheriting the same haplotype from both parents exhibits ROH [9]. McQuillan et al. [10] first used ROH to compute the genomic inbreeding coefficient (*F*_ROH_) in human. In animal genetics, ROH has been used to estimate whole-genome inbreeding at both the individual and population levels [11, 12] and to detect selection signatures [13–15]. Forutan et al. [7] reported that based on simulation *F*_ROH_ provides the closest estimates to true inbreeding [5]. Moreover, the correlations between *F*_PED_ and *F*_ROH_ were moderate to high (0.62–0.75) [16–18], so *F*_ROH_ was considered as an alternative approach to evaluate the inbreeding degree of an individual [5], particularly when pedigree information is unavailable or might be unreliable.

The length of IBD segments follows an inverse exponential distribution with a mean of 1/2 *t* Morgans, where *t* represents the number of generations from a common ancestor [19]. Therefore, the length of ROH can be used to infer inbreeding history. Shorter ROH are the result of more ancient inbreeding, while longer ROH suggest more recent inbreeding [9]. Hence, the detection and characterization of ROH can provide insight into how population history, structure and demography evolved over time [9, 17, 20] and to characterize inbreeding levels [15, 21]. Genomic regions enriched with ROH tend to generate ROH hotspots, which are also called ROH islands [9]. ROH islands could be used to positionally identify genes under natural or artificial selection in past adaptation and breeding processes [14, 15, 17, 22].

The aim of this study was to investigate the occurrence and distribution of ROH in five Chinese indigenous sheep breeds using genotypes assayed from the Ovine Infinium HD SNP BeadChip. These five sheep breeds are regionally disparate and possess breed specific characterizations. Based on ROH, we calculate genomic inbreeding coefficients and identify candidate genes residing in ROH islands in these Chinese indigenous sheep breeds.

## Materials and methods

### Animal populations and genotype quality control

Samples from a total of 450 sheep were collected representing five Chinese indigenous sheep breeds: Altay sheep (*n* = 50), Large-tailed Han sheep (*n* = 50), Hulun Buir sheep (*n* = 150), Short-tailed grassland sheep (*n* = 150) and Tibetan sheep (*n* = 50). Altay sheep were collected from Altay city in Xinjiang Uygur Autonomous Region, Large-tailed Han sheep from the national Large-tailed Han sheep conservation farm of China, Tibet sheep from Qinghai Tibet Plateau in Tianzhu county, Gansu Province, Hulun Buir sheep and Short-tailed grassland sheep from the grassland of Inner Mongolia Autonomous Region, China. All the animals were genotyped using an Ovine Infinium HD SNP BeadChip which included 604,715 SNPs. Genotype quality control was executed using PLINK v1.90 [23]. The following quality control criteria were used to filter the raw data: (1) locus call rate > 0.90; (2) minor allele frequency (MAF) > 0.01 and no evidence of Hardy Weinberg disequilibrium (*P* < 0.001); (3) SNPs located on autosomes; (4) call rate for individual > 90%. After quality control, 407 samples including 533,453 SNPs were retained for subsequent analyses. Chromosomal coordinates for each SNP were obtained from ovine genome assembly 3.1 (OAR3.1) (https://ftp.ncbi.nlm.nih.gov/genomes/refseq/vertebrate_mammalian/Ovis_aries/all_assembly_versions/suppressed/GCF_000298735.1_Oar_v3.1/). Missing genotypes were imputed using non pedigree methods in Beagle 5.0 software [24].

### Estimation of LD and effective population size

In this study, linkage disequilibrium (LD) coefficients (*r*^2^) between all pairwise SNPs separated less than 5 Mb in the genome were calculated for each breed using PLINK v1.09 software [23]. The mean *r*^2^ was calculated according to different pairwise distance classes as following [0 ~ ≤20; 20 ~ ≤40; 40 ~ ≤60;……; 4,940 ~ ≤4,960; 4,960 ~ ≤4,980; 4,980 ~ ≤5,000 kb].

Historical effective population sizes (*Ne*) of the five sheep breeds were computed as below by rearranging a formula proposed by Sved [25]:

1 $$ {N}_{e(t)}=\frac{1}{4{c}_t}\left(\frac{1}{E\left({r}^2|{c}_t\right)}-1\right) $$

where *N*_*e*(*t*)_ is the effective population size *t* generations prior to the genotyped animals and *t* is approximately equal to $\frac{1}{2c}$ [26]. The parameter *c* represents the genetic distance between two SNPs expressed in Morgans, such that *c* = 0.5 represents no linkage between two loci. We relate the sheep linkage map distances between pairwise markers from their physical locations on the same autosome according to the ratio of the total physical distance to the total recombinant genetic distance. The total physical distance and total genetic distance of sheep were obtained from the links https://ftp.ncbi.nlm.nih.gov/genomes/refseq/vertebrate_mammalian/Ovis_aries/all_assembly_versions/suppressed/GCF_000298735.1_Oar_v3.1/ and https://ftp.ncbi.nlm.nih.gov/genomes/MapView/Ovis_aries/non_sequence/, respectively. *E*(*r*^2^| *c*_*t*_) is the mean values of *r*^2^ between all pairwise SNPs spanning specific physical distance across all autosomes. In this study, the average ratio of the total physical distance to the total recombinant genetic distance was 1.415, and *c* = 0.1 M amounted to the average physical distance between SNP pairs of around 7.06 Mb, which can estimate *Ne* 5 generations previous to the genotyped animals. To better understand the historical change and trend of *Ne* for each breed, *Ne* of 1,000, 500, 200, 100, 50, 20, 10 and 5 generations ago were estimated, respectively.

### Identification of runs of homozygosity

The R package detectRUNS was used to detect ROH per individual [27]. The following criteria were set to detect ROH: (1) a sliding window of 50 SNPs; (2) a maximum of one heterozygous genotype per window; (3) the default value 0.05 as the threshold of the sliding window; (4) the maximum gap of 500 kb between two consecutive SNPs in ROH; (5) the minimum SNP density per ROH was set to one SNP every 50 kb; (6) the minimum ROH length was set to 500 Kb to exclude short ROH due to LD; (7) To minimize the number of ROH detected by chance, the minimum number of SNPs that constituted a ROH was set based on the method proposed by Lencz et al. [28]. That is:

2 $$ l=\frac{\log_e\left(\frac{\alpha }{n_{SNP}\times {n}_i}\right)}{\log_e\left(1-\overline{N_{het}}\right)} $$

where *l* is the minimum number of SNPs that must be in a ROH, *n*_*SNP*_ is the number of SNPs of each individual, *n*_i_ is the total number of individuals in the whole population, α is the false positive rate of identified ROH (set to 0.05 in the present study) and $\overline{N_{het}}$ was the mean heterozygosity of all SNPs. In this study, calculated from our genotypic data, *l* was equal to 53 SNPs.

### ROH size categories

For each sheep breed, the average number of ROH per individual and the average length of ROH were estimated. The identified ROH were divided into five classes based on their length: 0.5–5 Mb, 5–10 Mb, 10–15 Mb, 15–20 Mb and > 20 Mb. Frequency of the ROH numbers in each length category was calculated for the five sheep breeds. For each category, the mean sum of ROH per animal for each breed was calculated by summing all ROH in that category and averaging the sum number of animals in that breed. The ROH number of each chromosome for the five sheep breeds were counted respectively as well as the total length and total number of ROH for each animal.

### Estimation of ROH-based inbreeding coefficients

Genomic inbreeding coefficients based on ROH (*F*_ROH_) were computed for each individual using the equation proposed by McQuillan et al. [10]:

3 $$ {F}_{ROH}=\frac{\sum {L}_{ROH}}{L_{AUTO}} $$

where ∑*L*_*ROH*_ is the total length of all the ROH identified in an individual, *L*_*AUTO*_ is the total length of the autosomes covered by SNPs, which was 2,452 Mb in our study. To investigate differences of *F*_ROH_ on each chromosome, we calculated *F*_ROH_ for each chromosome. Moreover, inbreeding coefficient based on expected number of homozygous genotypes (*F*_HOM_) was calculated using PLINK v1.9 [23]. Pearson’s correlation between *F*_ROH_ and *F*_HOM_ was calculated.

### Detection of ROH islands

To identify the genomic regions most commonly associated with ROHs, the percentage of occurrence of SNPs in ROH was calculated by counting the number of times that a SNP was detected in an ROH across all the individuals in each breed. In this study, the top 0.1% of the SNPs observed in ROHs was selected as the threshold for identifying the genomic regions most commonly associated with ROH in each breed. A series of adjacent SNPs that exceeded this threshold formed a genomic region which we refer to as an ROH island. In this study, the breed specific thresholds were 40%, 44%, 37%, 39% and 50% of the individuals sharing the overlapping homozygous regions (ROH islands) in Altay sheep, Large-tailed Han sheep, Hulun Buir sheep, Short-tailed grassland sheep and Tibetan sheep, respectively. Gene annotation in ROH islands was performed on the basis of sheep reference genome Ovis_aries.Oar_v3.1. The biological function of the genes residing in ROH islands was conducted by survey of relevant literature.

## Results

### Estimation of LD and effective population size (*Ne*)

**Fig. 1:**
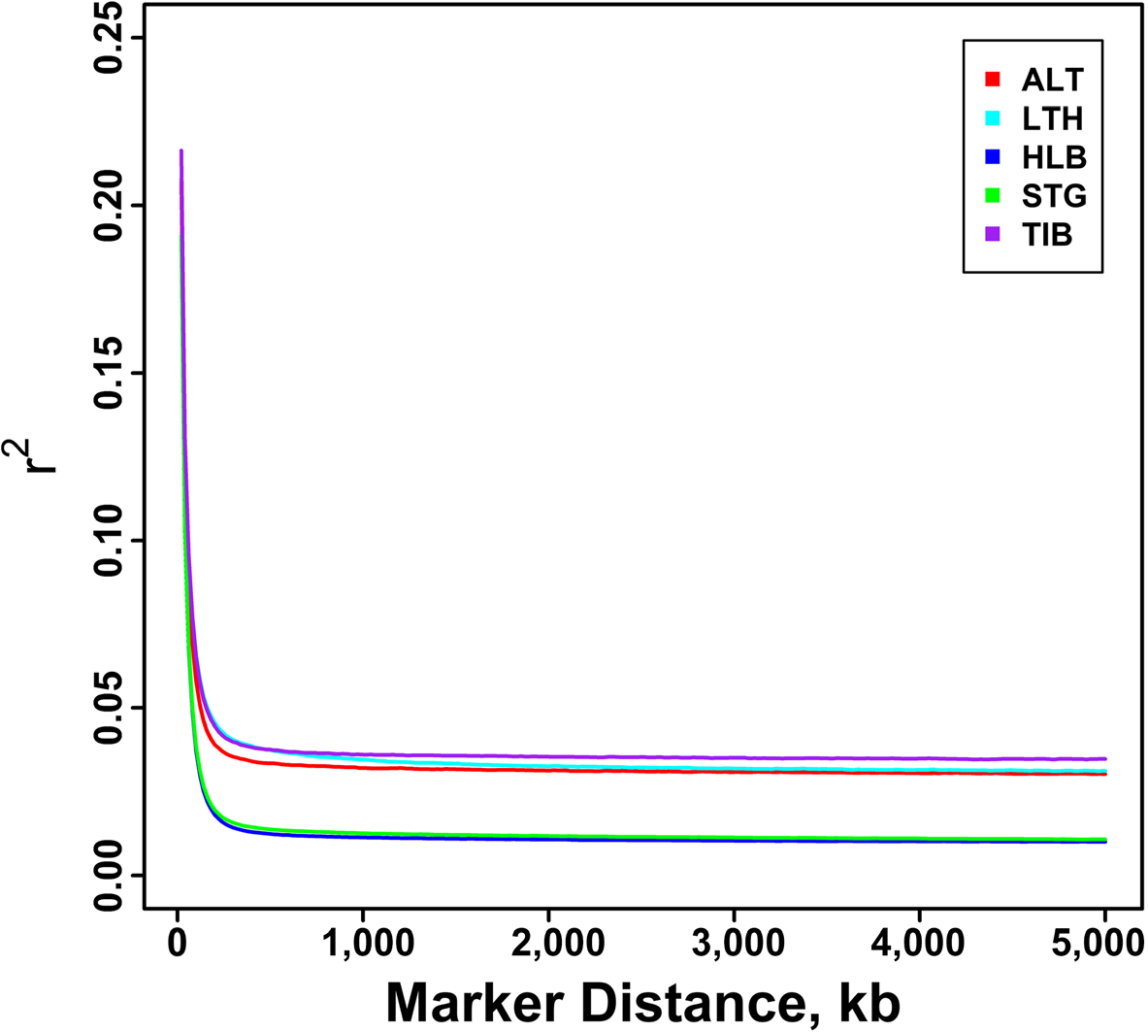
LD decay map measured by *r*^2^ over distance between SNPs in five Chinese indigenous sheep breeds. ALT, LTH,HLB, STG and TIB represent Altay sheep, Large-tailed Han sheep, Hulun Buir sheep, Short-tailed grassland  sheep and Tibetan sheep, respectively

Figure 1 shows the average *r*^2^ per breed plotted against the physical distances between pairwise SNPs in classes of 20 kb, providing an overview of the decline of *r*^2^ in each breed. On the whole, Tibetan sheep showed the highest average *r*^2^ at all marker distances, followed by Large-tailed Han sheep and Altay sheep. The average *r*^*2*^ of Hulun Buir sheep and Short-tailed grassland sheep decayed significantly faster than *r*^*2*^ of the other breeds. Moreover, LD decay lines of Hulun Buir sheep and Short-tailed grassland sheep almost overlapped but the smallest values of average *r*^2^ were apparent in Hulun Buir sheep.

**Fig. 2:**
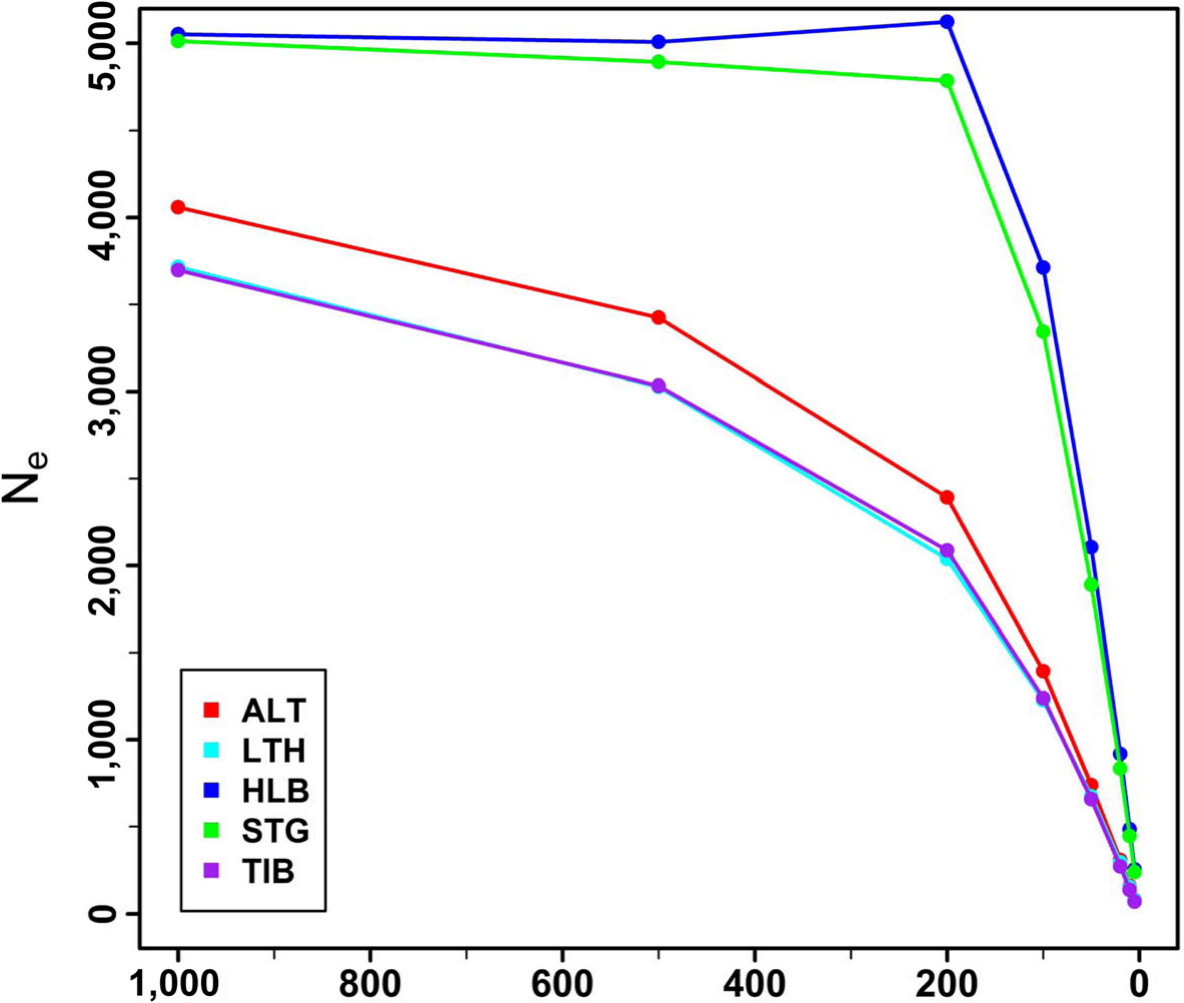
Estimates of effective population sizes (Ne) for Chinese five sheep breeds for 1000–5 ancestral generations ago. ALT, LTH, HLB, STG and TIB represent Altay sheep, Large-tailed Han sheep, Hulun Buir sheep, Short-tailed grassland sheep and Tibetan sheep, respectively

Estimates of effective population size (*Ne*) for the five sheep breeds are depicted in Fig. 2. For all five breeds, a declining trend of effective population size (*Ne*) across generations was observed. For all generations, Hulun Buir sheep and Short-tailed grassland sheep had larger *Ne*, relative to other  sheep breeds. The *Ne* of the five sheep breeds at 1,000 generations ago were predicted to be 5,053 (Hulun Buir sheep), 5,013 (Short-tailed grassland sheep), 4,059 (Altay sheep), 3,715 (Large-tailed Han sheep) and 3,697 (Tibetan sheep). In a more recent time frame (5 generations ago), the corresponding estimates of *Ne* were 253 (Hulun Buir sheep), 237 (Short-tailed grassland sheep), 81 (Altay sheep), 78 (Large-tailed Han sheep) and 70 (Tibetan sheep). The sequences of estimated effective population sizes by generation and breed are shown in Table S1.

### ROH detection

**Fig. 3:**
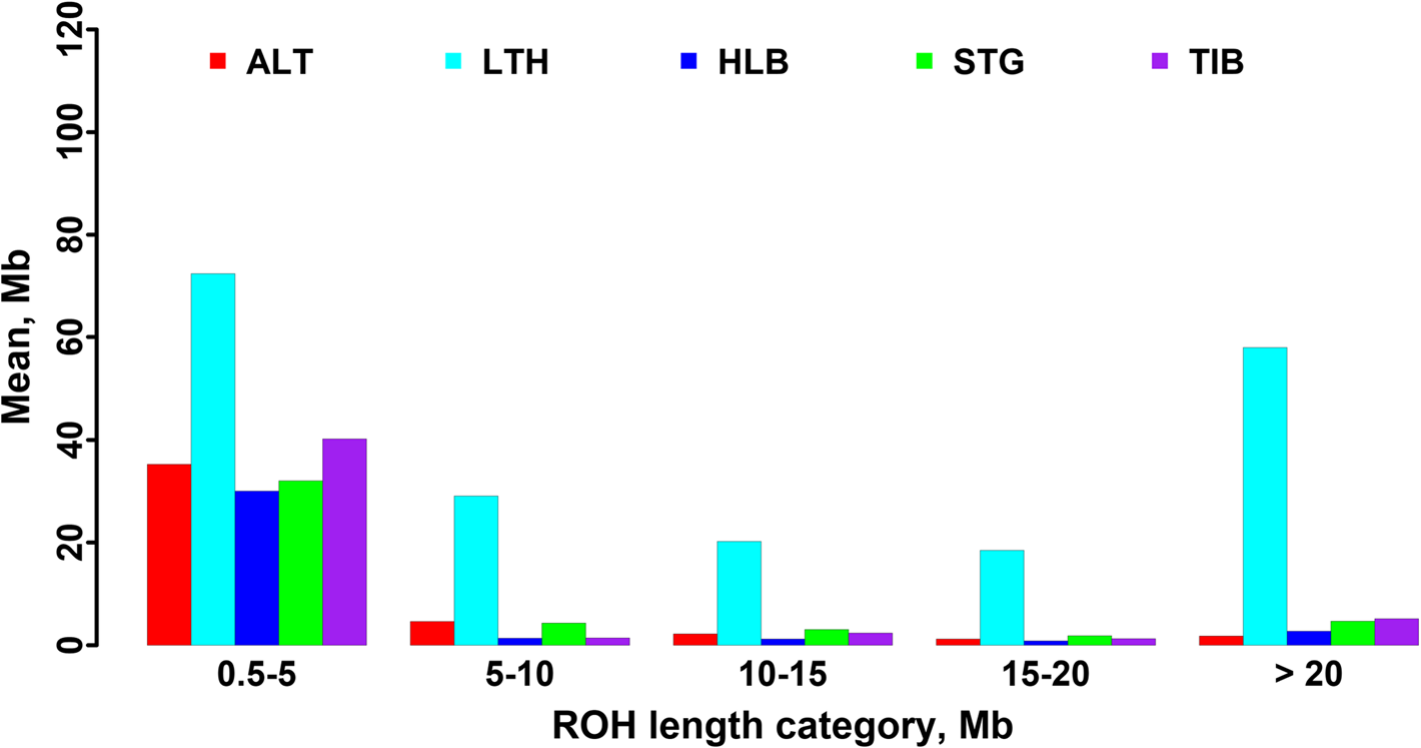
The Mean sum of ROH in Mb per animal within each ROH length category. ALT, LTH, HLB, STG and TIB represent Altay sheep, Large-tailed Han sheep, Hulun Buir sheep, Short-tailed grassland sheep and Tibetan sheep, respectively

**Fig. 4:**
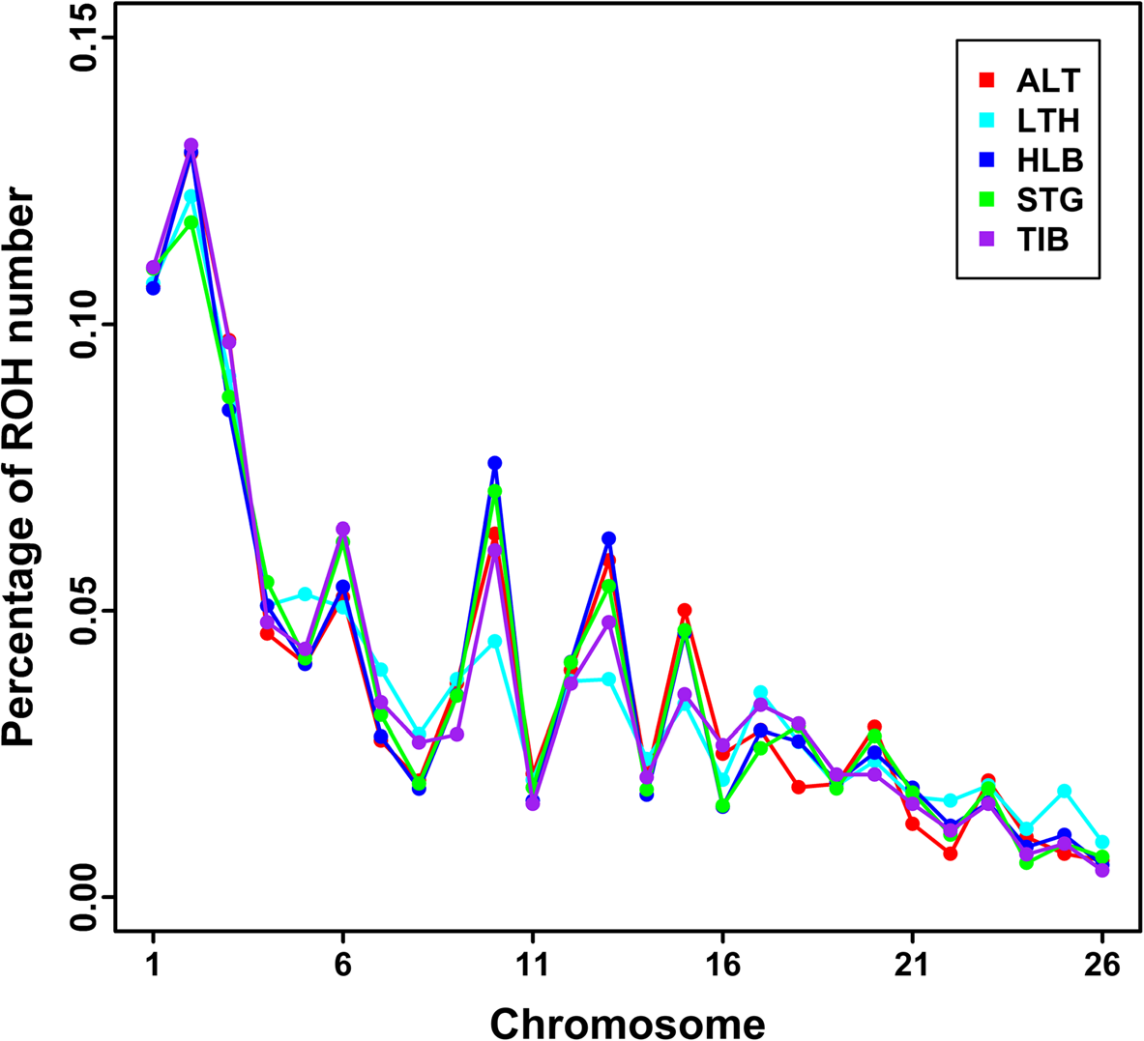
Number of ROH per chromosome in five sheep breeds. ALT, LTH, HLB, STG and TIB represent Altay sheep, Large-tailed Han sheep, Hulun Buir sheep, Short-tailed grassland sheep and Tibetan sheep, respectively

**Fig. 5:**
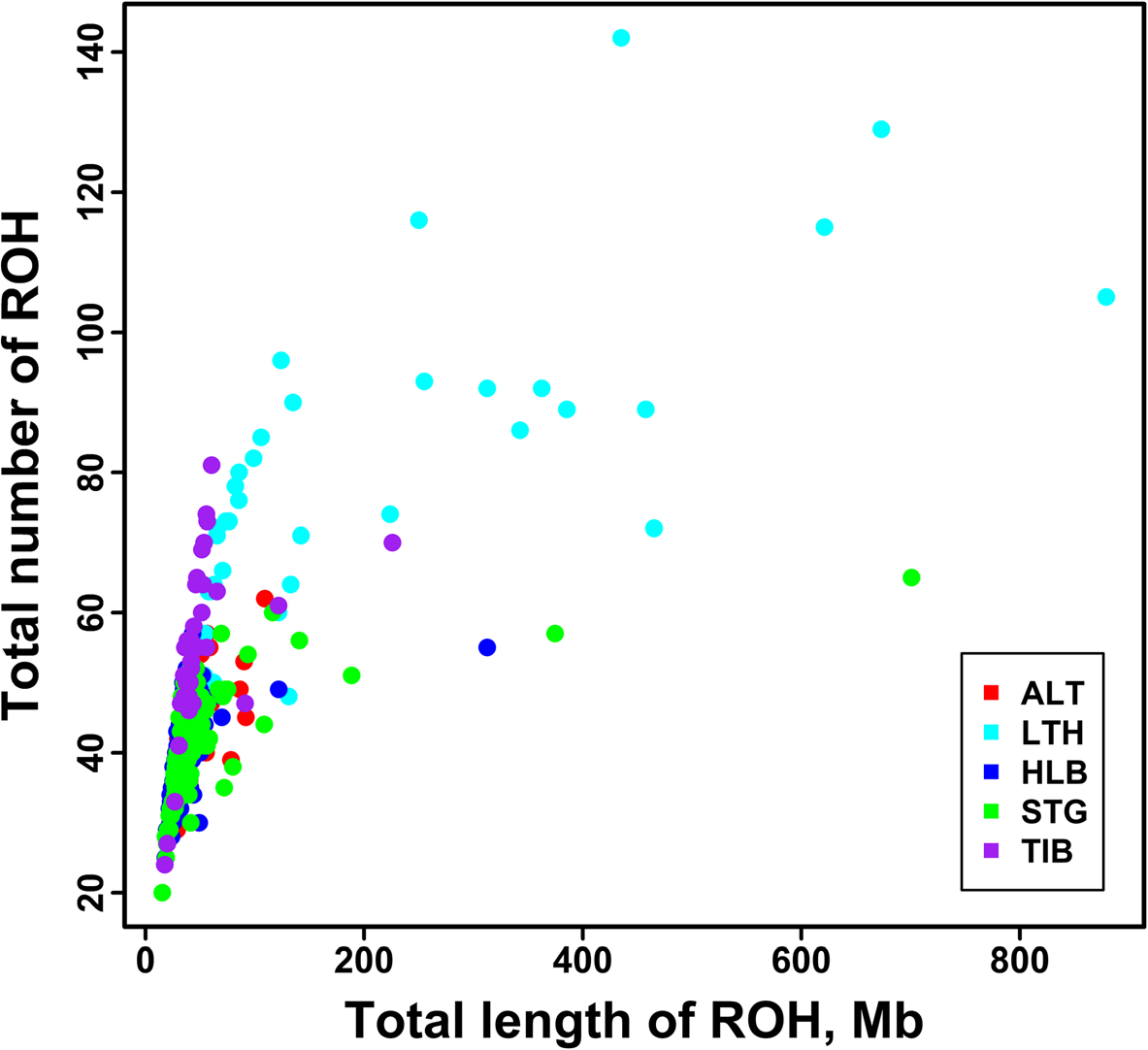
Total number of ROHs and total length of genome (Mb) covered by ROH segments per individual for each sheep breed. ALT, LTH, HLB, and TIB represent Altay sheep, Large-tailed Han sheep, Hulun Buir sheep, Short-tailed grassland sheep and Tibetan sheep, respectively

A total of 18,288 ROHs were identified across the five sheep breeds. Table 1 summarizes the average number of ROH per individual and the average length of ROH per sheep breed. The average number of ROH per individual ranged from 78 (Large-tailed Han sheep) to 39 (Hulun Buir sheep), and the average length of ROH each breed ranged from 0.929 Mb (Hulun Buir sheep) to 2.554 Mb (Large-tailed Han sheep). Table 2 shows the percentages of ROH numbers in five length categories of 0.5–5, 5–10, 10–15, 15–20 and > 20 Mb in each breed. Regardless of breed, most ROH were shorter than 5 Mb. Compared with the other four sheep breeds, Large-tailed Han sheep had a higher proportion of long ROHs (> 5 Mb). Fig. 3 illustrates the mean sum of ROH in each length category of the five sheep breeds. Large-tailed Han had the highest mean sum of ROH in all ROH length categories. Especially in the category of > 20 Mb, the gap between Large-tailed Han and other breeds was more prominent. As seen in Fig. 4, the percentages of ROH numbers on autosomes varied but the trends across the five sheep breeds tended to be similar. The highest percentage was observed on OAR2 in all five sheep breeds, while the lowest percentage of ROH number was on OAR24 in Short-tailed grassland sheep and OAR26 in the other four breeds. On the whole, the numbers of ROH per chromosome tended to increase with chromosome length, with the average correlation coefficient of 0.934 across all sheep breeds. Figure 5 depicts the total number and the total length of ROH per individual. Several extreme individuals exhibiting autosomal ROH > 600 Mb were found in Large-tailed Han sheep and Short-tailed grassland sheep breeds.

**Table 1:** Descriptive statistics for ROH and genomic inbreeding coefficients in five sheep breeds

Breeds	ROH number	ROH length, Mb	*F*_ROH_	*F*_HOM_	${r}_{\left({F}_{ROH}-{F}_{HOM}\right)}$
	Mean ± SD	Mean ± SD	Mean ± SD	Mean ± SD	
ALT	42.95 ± 7.52	1.053 ± 1.709	0.0184 ± 0.0081	−0.0121 ± 0.00865	0.868
LTH	77.56 ± 22.90	2.554 ± 5.647	0.0808 ± 0.0810	0.0444 ± 0.0847	0.997
HLB	39.08 ± 6.10	0.929 ± 1.988	0.0148 ± 0.0139	−0.00334 ± 0.0157	0.909
STG	40.08 ± 7.31	1.145 ± 2.386	0.0187 ± 0.0266	−0.000860 ± 0.0286	0.950
TIB	53.70 ± 11.85	0.939 ± 1.951	0.0206 ± 0.0138	−0.0163 ± 0.0250	0.721
ALL	44.93 ± 15.25	1.278 ± 3.055	0.0234 ± 0.0364	0.0129 ± 0.0403	0.960

Note: *ALT*, *LTH*, *HLB*, *STG* and *TIB* represent Altay sheep, Large-tailed Han sheep, Hulun Buir sheep, Short-tailed grassland sheep and Tibetan sheep, respectively

**Table 2:** The percentages of ROH number in different length categories in the five sheep breeds

Categories, Mb	ALT	LTH	HLB	STG	TIB
0.5–5	0.977	0.888	0.989	0.972	0.988
5–10	0.016	0.054	0.005	0.015	0.004
10–15	0.004	0.021	0.003	0.006	0.004
15–20	0.002	0.014	0.001	0.003	0.001
> 20	0.002	0.023	0.002	0.004	0.003

Note: *ALT*, *LTH*, *HLB*, *STG* and *TIB* represent Altay sheep, Large-tailed Han sheep, Hulun Buir sheep, Short-tailed grassland sheep and Tibetan sheep, respectively

### Genomic inbreeding coefficients

**Fig. 6:**
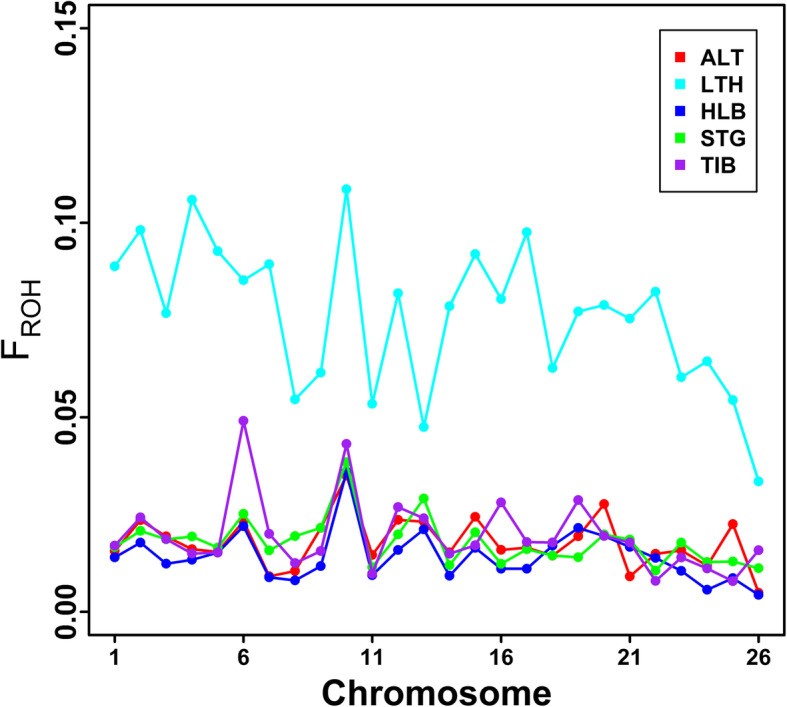
Distribution of *F*_ROH_ on each *Ovies aries* chromosome (OAR) in five sheep breeds. ALT, LTH, HLB, STG and TIB represent Altay sheep, Large-tailed Han sheep, Hulun Buir sheep, Short-tailed grassland sheep and Tibetan sheep, respectively

Table 1 shows the two measures of inbreeding (*F*_ROH_ and *F*_HOM_) in the five sheep breeds. All the average *F*_ROH_ of the five sheep breeds were bigger than 0.01, while the average *F*_HOM_ in Altay sheep, Hulun Buir sheep, Short-tailed grassland sheep and Tibetan sheep breeds were negative. The average of *F*_ROH_ of Hulun Buir sheep was the lowest (0.0148) among these five sheep breeds, whereas the average *F*_ROH_ of Large-tailed Han sheep was the highest (0.0808). It should be noted that the *F*_ROH_ of Short-tailed grassland sheep was very close to Hulun Buir sheep breed. The correlations between *F*_ROH_ and *F*_HOM_ ranged from 0.721 (Tibetan sheep) to 0.997 (Large-tailed Han sheep) in five sheep breeds, and the correlation coefficient across all the animals was 0.960. The *F*_ROH_ per chromosome per breed are illustrated in Fig. 6. The autosomal values of *F*_ROH_ of Large-tailed Han sheep were the highest across all the five breeds.

### Detection of common ROHs

**Fig. 7:**
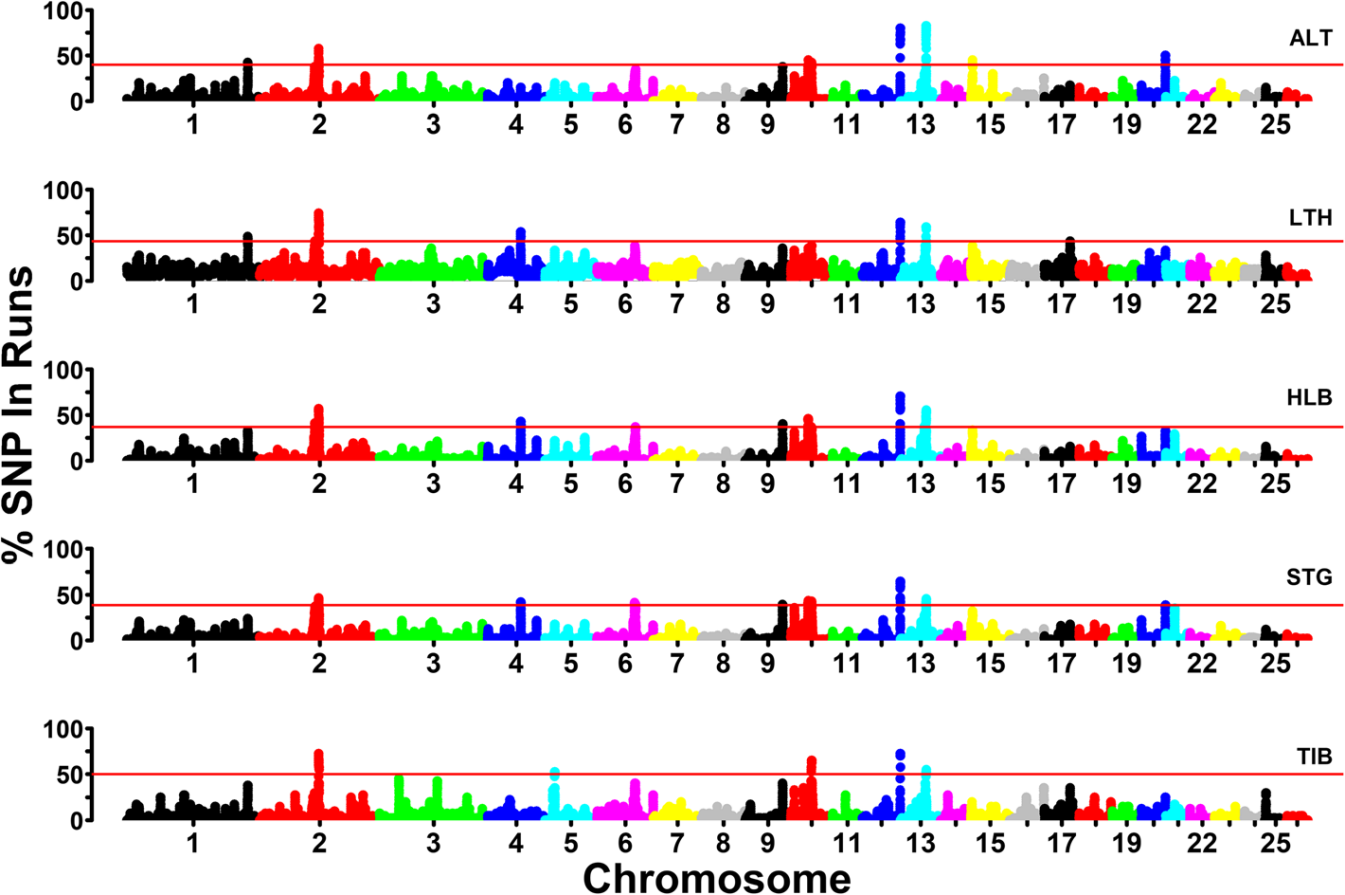
Genome-wide frequency of SNPs occurrence into ROHs for each sheep breed. The red line was the threshold to define the ROH islands. ALT, LTH, HLB, STG and TIB represent Altay sheep, Large-tailed Han sheep, Hulun Buir sheep, Short-tailed grassland sheep and Tibetan sheep, respectively

Fig. 7 displays the percentage of occurrence of SNPs in ROH against the position of the SNP along all the autosomes. As seen in Fig. 7, ROH islands were mainly distributed on OARs 2, 9, 10, 12 and 13, with many overlap regions observed among the five sheep breeds. Totally, 49 genomic regions were identified as ROH islands in the five sheep breeds (Table 3). Three of those genomic regions were common to all the five breeds. They were located on OAR2: 12.2–12.3 Mb, OAR12: 78.4–79.1 Mb and OAR13: 53.0–53.6 Mb. In addition, there were three breed-specific ROH islands in Altay sheep (OAR15: 3.4–3.8 Mb), Large-tailed Han sheep (ORA17: 53.5–53.8 Mb) and Tibetan sheep (ORA5:19.8–20.2 Mb). From the genomic regions representing ROH islands in the five sheep breeds, a total of 257 positional candidate genes were annotated. After removing genes that were represented more than once, 76 unique genes remained. Among them, 19 genes were reported in the literature as having been associated with economically important traits (Table 4), whereas the other genes are listed in Table S2.

**Table 3:** List of ROH islands identified in five Chinese indigenous sheep breeds

Breeds	Chr	Number of SNPs	Start, bp	End, bp	Number of genes
ALT	1	61	250,505,889	250,968,614	2
	2	29	122,022,456	122,196,859	0
	2	67	122,203,171	122,713,621	1
	2	49	122,789,438	123,131,444	0
	10	1	36,431,208	36,431,208	1
	10	12	42,602,855	42,668,804	0
	10	2	42,864,819	42,886,791	0
	10	2	43,201,824	43,218,840	0
	12	79	78,441,984	79,070,188	7
	13	104	52,983,990	53,669,096	27
	15	71	3,369,761	3,860,098	1
	20	53	49,963,739	50,507,014	1
LTH	1	68	250,505,243	251,024,337	2
	2	9	114,531,332	114,582,444	0
	2	188	122,066,517	123,448,890	1
	4	86	68,604,130	69,128,428	21
	12	91	78,412,601	79,070,188	7
	13	90	53,019,664	53,640,527	25
	17	33	53,546,799	53,758,306	2
HLB	2	3	114,602,806	114,611,225	0
	2	19	115,006,350	115,133,173	0
	2	146	122,203,171	123,318,733	1
	4	59	68,730,153	69,128,428	21
	6	1	79,981,634	79,981,634	0
	9	61	77,276,731	77,807,912	1
	10	105	35,838,530	36,431,208	10
	10	12	42,602,855	42,668,804	0
	12	79	78,441,984	79,070,188	7
	13	4	49,772,494	49,800,472	0
	13	82	53,046,392	53,647,951	25
STG	2	67	122,203,171	122,713,621	1
	4	55	68,730,153	69,082,247	20
	6	3	78,164,118	78,171,900	0
	6	22	78,190,079	78,372,681	1
	6	5	79,989,614	80,013,968	0
	6	41	80,045,125	80,283,293	0
	9	43	77,387,147	77,790,278	1
	10	47	35,839,462	36,132,909	5
	10	52	36,173,170	36,431,208	6
	10	34	42,602,855	42,829,373	0
	10	29	42,862,671	43,163,671	0
	12	78	78,449,224	79,070,188	7
	13	72	53,065,617	53,589,429	22
	20	7	50,349,355	50,424,219	1
TIB	2	154	122,210,623	123,369,957	1
	5	75	19,764,108	20,233,040	4
	10	168	42,182,526	43,525,344	0
	12	78	78,449,224	79,070,188	7
	13	55	53,152,803	53,589,429	18

Note: ALT, LTH, HLB, STG and TIB represent Altay sheep, Large-tailed Han sheep, Hulun Buir sheep, Short-tailed grassland sheep and Tibetan sheep, respectively

**Table 4:** Candidate genes resided in ROH island associated with economic traits of animals

Breeds	OAR	Position, bp	Candidate genes	Gene function
ALT, LTH	1	250,958,731 ~ 251,069,283	*PCCB*	Growth and carcass
LTH, HLB,STG	4	68,858,042 ~ 68,863,494	*HOXA10*	Fat deposition
LTH, HLB, STG	4	68,921,977 ~ 68,924,549	*HOXA3*	Embryo development
TIB	5	19,737,330 ~ 19,769,293	*P4HA2*	Hypoxic adaptation
TIB	5	19,956,144 ~ 19,958,155	*CSF2*	Immunity and inflammation response
TIB	5	19,971,457 ~ 19,973,243	*IL3*	Immunity regulation
HLB, STG	10	35,862,425 ~ 35,885,746	*LATS2*	Embryonic development
HLB, STG	10	36,045,326 ~ 36,103,818	*IFT88*	Inflammatory response
HLB, STG	10	36,253,000 ~ 36,253,785	*GJB6*	Body size and development
HLB, STG	10	36,271,774 ~ 36,272,454	*GJB2*	Body size and development
HLB, STG	10	36,304,573 ~ 36,305,769	*GJA3*	Body size and development
ALT, LTH, HLB, STG, TIB	12	78,543,637 ~ 78,552,280	*CSRP1*	Growth and carcass
ALT, LTH, HLB, STG, TIB	12	78,591,681 ~ 78,596,742	*TNNI1*	Growth, carcass and meat quality
ALT, LTH, HLB, STG	13	53,097,296 ~ 53,098,312	*NPBWR2*	Reproductive activity
ALT, LTH,HLB, STG, TIB	13	53,280,623 ~ 53,282,184	*ABHD16B*	Male infertility
ALT, LTH, HLB, STG, TIB	13	53,482,080 ~ 53,488,365	*EEF1A2*	Muscle development and lipid metabolism
LTH	15	3,848,546 ~ 4,133,998	*PDGFD*	Lipid metabolism
LTH	17	53,560,417 ~ 53,616,466	*P2RX7*	Final weight and backfat thickness
LTH	17	53,661,638 ~ 53,752,199	*IFT81*	Spermiogenesis and fertility

Note: *ALT*, *LTH*, *HLB*, *STG* and *TIB* represent Altay sheep, Large-tailed Han sheep, Hulun Buir sheep, Short-tailed grassland sheep and Tibetan sheep, respectively

## Discussion

**Linkage disequilibrium (LD) and effective population size (*Ne*) affected by demography**

In this study, we collected five Chinese indigenous sheep breeds with different tail types: short fat-tailed (Short-tailed grassland sheep), medium fat-tailed (Hulun Buir sheep), long fat-tailed (Large-tailed Han sheep), fat-rumped (Altay sheep), and thin-tailed sheep (Tibetan sheep). Large-tailed Han sheep possess the fattiest and largest tails of all Chinese local sheep breeds. The conspicuous feature of Large-tailed Han sheep is their long fat tails, which can reach the ground in some extreme individuals. A remarkable feature of Altay sheep is their fat buttocks. Among all the sheep breeds, the highest value of average *r*^2^ was observed in Tibetan sheep, which had the smallest *Ne*. LD decay lines of Hulun Buir sheep and Short-tailed grassland sheep almost overlapped and the smallest values of average *r*^2^ were showed in Hulun Buir sheep. Short-tailed grassland sheep and Hulun Buir sheep are distributed in Hulun Buir grassland in the Inner Mongolia Autonomous Region [29]. Their *Ne* were about 253 and 238 at five generation ago, respectively, which were close to *Ne* of Sunite sheep (207) at seven generations ago in our previous study [30]. Like Hulun Buir sheep and Short-tailed grassland sheep, Sunite sheep also originated from Mongolia sheep and had a similar breed history and management system. These results demonstrate the high genetic diversity of Mongolian sheep.

### ROH and ROH-based inbreeding coefficient (*F*_ROH_)

Since the length of ROH can be used to infer when inbreeding happened, the number, length and distribution of ROH can provide valuable information about the demography history. Furthermore, we can further utilize the lengths of ROH to estimate the ROH-based inbreeding coefficients. In the current study, ROH identified in all five sheep breeds were unevenly distributed (Fig. 4), with OAR2 having the largest number of ROH among all sheep populations. The number of ROH had high positive correlation with chromosome length (0.934). Our results were consistent with other sheep breeds [31, 32]. However, the smallest number of ROH per chromosome was on different chromosomes in different sheep breeds [31, 32]. Moreover, the mean numbers of ROH varied in the five sheep breeds as well as the average lengths of ROH. Among these breeds, Large-tailed Han sheep had the highest average number of ROH per animal (77.56), the longest average length of ROH (2.554 Mb), and the highest proportion of long ROH fragments (> 5 Mb), especially ROH > 20 Mb (Fig. 3). Moreover, the most individuals carrying a large number of ROH with a total length ≥ 600 Mb were mainly observed in Large-tailed Han sheep. Furthermore, Large-tailed Han sheep showed the highest *F*_ROH_ in both genome (0.0808) and chromosome level (Fig. 7). These results demonstrate that Large-tailed Han sheep had low genetic diversity, and more recent inbreeding events. This might be due to the uncontrolled mating of related individuals in the national Large-tailed Han sheep conservation of China where we sampled these individuals. On the contrary, the Hulun Buir sheep breeds exhibited the least mean number of ROH per animal (39.08) and the shortest average length of ROH (0.929 Mb). Hulun Buir sheep also showed the lowest *F*_ROH_, followed by Short-tailed grassland sheep which was consistent with the results of effective population size. These reflected their low level of inbreeding resulting from management systems based on random mating in the grassland. The difference of mean number per animal and average length of ROH may reflect the demography of the different populations. In general, the results of ROH had reflected the inbreeding and population history of the five sheep breeds, and the results of LD and effective population size basically supported and verified the results of ROH. Our results seemed to indicate that ROH can be used as a useful tool for inbreeding evaluation and livestock conservation.

### Candidate genes within ROH islands

ROH islands are generated from natural or artificial selection and could be used to identify selection signatures. In the process of long-term domestication and adaptation, sheep breeds have formed breed-specific traits. The high frequency homozygous fragments in the genome representing ROH islands can be used to elucidate the genetic mechanism of the breed specific traits. The thresholds in the present study were more stringent than those of other studies using low-density chips [12, 13], which could avoid false positive results.

There were three breed-specific ROH islands: in Altay sheep, Large-tailed Han sheep and Tibetan sheep. In Altay sheep, the specific ROH island was located on OAR15: 3.4–3.8 Mb. In that genomic region, the fat-tail sheep breeds (Large-tailed Han sheep, Hulun Buir sheep and Short-tailed grassland sheep) also had peaks close to the top 0.1% threshold line (Fig. 7). This genomic region harbors *PDGFD* that has been documented as a causal gene for fat deposition in sheep tails [33–37]. Moreover, *HOXA10* was identified in overlapped ROH island (OAR4: 68.7–69.1 Mb) of Hulun Buir sheep, Short-tailed grassland sheep and Large-tailed Han sheep populations. *HOXA10* was identified as a candidate gene related to tail type by selection signature detection [35] and further validated as a candidate gene strongly linked with fat deposition in sheep tail by RNA Seq [38]. In addition, *PCCB* resided in the overlapped ROH islands of Large-tailed Han sheep and Altay sheep, and is involved in the metabolism of fatty acids in pig [39].

These results were supported by the samples with obvious breed feature in terms of tail types. According to sheep tail morphology, the five sheep breeds can be classified into five classes: long fat-tailed (Large-tailed Han sheep), median fat-tailed (Hulun Buir sheep), short fat-tailed (Short-tailed grassland sheep), fat-rumped (Altay sheep) or thin-tailed sheep (Tibetan sheep). In the Tibetan sheep population, the breed specific ROH island resided in OAR5: 19.8–20.2 Mb. That genomic region harbored *P4HA2*, which is related to hypoxic adaptation and can be induced to express in hypoxic conditions [40, 41]. This may indicate that *P4HA2* gene had been selected in the process of Tibetan sheep adapting to high altitude environment. In Large-tailed Han sheep population, the breed specific ROH island was on the OAR17: 53.5–53.8 Mb. On that region, *P2RX7* was also annotated, and that gene had been found to be associated with the final weight and backfat thickness of Landrace pigs [42].

Three ROH islands located on OAR2: 12.2–12.3 Mb, OAR12: 78.4–79.1 Mb and OAR13: 53.0–53.6 Mb were common to all the five sheep breeds. The latter two genomic regions harbored four important candidate genes of *TNNI1*, *CSRP1*, *EEF1A2* and *ABHD16B*. *TNNI1* has been implicated with carcass, growth and meat quality traits in pigs [43, 44] and cattle [45]. *CSRP1* was identified as a strong candidate gene associated with growth and carcass traits through SNV and haplotype analysis in the Chinese beef cattle [46]. *EEF1A2* was involved in muscle development and lipid metabolism during fetal development in sheep [47]. Furthermore, *GJB2*, *GJB6* and *GJA3* were found in overlapping ROH islands of Hulun Buir sheep and Short-tailed grassland sheep, and have documented associations with body size and development by selection signature detection of Egyptian sheep and goat populations [48]. *ABHD16B* is the potential causative protein-altering variant for male infertility in Holstein cattle [49]. Other genes have documented involvement in reproduction. *IFT81* was identified from ROH island in Large-tailed Han sheep population, and played an essential role in spermiogenesis and fertility male mice [50]. *NPBWR2* was located on the overlapped ROH islands in Altay sheep, Hulun Buir sheep, Short-tailed grassland sheep and Large-tailed Han sheep, and play a role in modulating the reproductive activity in the pig [51]. *HOXA3* resided in the overlapped ROH island in Hulun Buir sheep, Short-tailed grassland sheep and Large-tailed Han sheep populations and was reported to be expressed in bovine oocytes and early-stage embryos and may influence oocyte maturation and the first stages of embryonic development [52]. *LATS2* was located on the overlapped ROH islands in Hulun Buir sheep and Short-tailed grassland sheep, and plays an essential role in embryonic development, proliferation control and genomic integrity [53].

In addition, we identified several genes related to immune and inflammatory response. We identified *CSF2* and *IL3* from the ROH islands of Tibetan sheep. Previous study had shown that *CSF2* played an important role in immunity regulation, hematopoiesis and inflammation response [54–56]. Furthermore, *CSF2* was also reported that played pivotal roles in implantation events during early pregnancy in pigs [57] and influence the reproductive capacity in mice [58]. *IFT88* resided in the overlapped ROH islands from Hulun Buir sheep and Short-tailed grassland sheep and had been reported to be involved in the inflammatory response of interleukin-1 [59].

## Conclusions

In this study, we used genotypes assayed using an Ovine Infinium HD SNP BeadChip to characterize the pattern of LD, estimate the effective population sizes and investigate the occurrence and distribution of ROH across the genomes of five Chinese indigenous sheep breeds. Different LD and ROH patterns were observed in the five breeds. The large-tailed Han sheep population had the highest genomic inbreeding coefficients and the highest proportion of long ROH fragments which reflect recent inbreeding events. On the contrary, the opposite conditions were present in Hulun Buir sheep. In total, 49 ROH islands were identified. Three ROH islands were common to all the breeds, and three breed-specific ROH islands were in Altay sheep, Large-tailed Han sheep and Tibetan sheep. These ROH islands harbored 78 unique genes, including 19 genes documented as being involved in tail types, adaptation, growth, body size, reproduction or immune response. Our findings contribute to the understanding of genetic diversity, population demography and the underlying genetic mechanism of economically important traits, and help design and implement breeding and conservation strategies for Chinese sheep.

## Supplementary Information

**Additional file 1: Table S1** The effective population size across generations for each breed. **Table S2** ROH hotspots identified in five Chinese indigenous sheep breeds and candidate genes annotated.

## Data Availability

The genotypic data was available at figshare: 10.6084/m9.figshare.14524332.
