# Supplementary material for: Estimates of genomic inbreeding and identification of candidate regions that differ between Chinese indigenous sheep breeds
Source: J Anim Sci Biotechnol. 2021 Aug 5;12:95. doi: 10.1186/s40104-021-00608-9 (PMC8340518; doi:10.1186/s40104-021-00608-9)
Supplement: Supplementary file 1 — Additional file 1: Table S1 The effective population size across generations for each breed. Table S2 ROH hotspots identified in five Chinese indigenous sheep breeds and candidate genes annotated. [file 40104_2021_608_MOESM1_ESM.docx]

Table S1 The effective population size across generations for each population

| **Generations(ago)** | **ALT** | **LTH** | **BHL** | **SHL** | **TIB** |
| --- | --- | --- | --- | --- | --- |
| 5 | 81 | 78 | 253 | 238 | 70 |
| 10 | 158 | 152 | 484 | 444 | 138 |
| 20 | 307 | 294 | 917 | 833 | 270 |
| 50 | 738 | 674 | 2105 | 1889 | 657 |
| 100 | 1393 | 1225 | 3712 | 3343 | 1239 |
| 200 | 2391 | 2038 | 5123 | 4784 | 2086 |
| 500 | 3424 | 3028 | 5008 | 4893 | 3033 |
| 1000 | 4059 | 3715 | 5053 | 5013 | 3697 |

**Table S2 ROH hotspots identified in five Chinese indigenous sheep populations and candidate genes annotated**

| Populations | Chr | Number of SNPs | Start bp | End bp | No.  genes | Gene names |  |
| --- | --- | --- | --- | --- | --- | --- | --- |
| ALT | 1 | 61 | 250505889 | 250968614 | 2 | *STAG1, PCCB* |  |
|  | 2 | 29 | 122022456 | 122196859 | 0 | 0 |  |
|  | 2 | 67 | 122203171 | 122713621 | 1 | *FSIP2* |  |
|  | 2 | 49 | 122789438 | 123131444 | 0 | 0 |  |
|  | 10 | 1 | 36431208 | 36431208 | 1 | *ZMYM5* |  |
|  | 10 | 12 | 42602855 | 42668804 | 0 | 0 |  |
|  | 10 | 2 | 42864819 | 42886791 | 0 | 0 |  |
|  | 10 | 2 | 43201824 | 43218840 | 0 | 0 |  |
|  | 12 | 79 | 78441984 | 79070188 | 7 | *CSRP1, TNNI1, NAVI, IP09, SHISA4, RNPEP, ELF3* |  |
|  | 13 | 104 | 52983990 | 53669096 | 27 | *PCMTD2, MYT1, NPBWR2, OPRL1, RGS19, TCEA2, PRPF6, RF00026, ZNF512B, UCKL1, DNAJC5, ABHD16B, ZBTB46, ZGPAT, ARFRP1, TNFRSF6B, STMN3, GMEB2, FNDC11, SRMS, PTK6, EEF1A2, KCNQ2, CHRNA4, ARFGAP1, BIRC7, YTHDF1* |  |
|  | 15 | 71 | 3369761 | 3860098 | 1 | *PDGFD* |  |
|  | 20 | 53 | 49963739 | 50507014 | 1 | *GMDS* |  |
| LTH | 1 | 68 | 250505243 | 251024337 | 2 | *STAG1, PCCB* |  |
|  | 2 | 9 | 114531332 | 114582444 | 0 | 0 |  |
|  | 2 | 188 | 122066517 | 123448890 | 1 | *FSIP2* |  |
|  | 4 | 86 | 68604130 | 69128428 | 21 | *EVX1, RF02043, RF02042, RF02041, RF02040, RF02142, RF02141, RF02140, RF02139, RF02138, RF02137, HOXA10, HOXA6, HOXA3, HOXA2, RF01979, RF01978, RF01976, RF01975, HOXA1, SKAP2* |  |
|  | 12 | 91 | 78412601 | 79070188 | 7 | *CSRP1, TNNI1, NAVI, IP09, SHISA4, RNPEP, ELF3* |  |
|  | 13 | 90 | 53019664 | 53640527 | 25 | *MYT1, NPBWR2, OPRL1, RGS19, TCEA2, PRPF6, RF00026, ZNF512B, UCKL1, DNAJC5, ABHD16B, ZBTB46, ZGPAT, ARFRP1, TNFRSF6B, STMN3, GMEB2, FNDC11, SRMS, PTK6, EEF1A2, KCNQ2, CHRNA4, ARFGAP1, BIRC7* |  |
|  | 17 | 33 | 53546799 | 53758306 | 2 | *P2RX7, IFT81* |  |
| BHL | 2 | 3 | 114602806 | 114611225 | 0 | 0 |  |
|  | 2 | 19 | 115006350 | 115133173 | 0 | 0 |  |
|  | 2 | 146 | 122203171 | 123318733 | 1 | *FSIP2* |  |
|  | 4 | 59 | 68730153 | 69128428 | 21 | *EVX1, RF02043, RF02042, RF02041, RF02040, RF02142, RF02141, RF02140, RF02139, RF02138, RF02137, HOXA10, HOXA6, HOXA3, HOXA2, RF01979, RF01978, RF01976, RF01975, HOXA1, SKAP2* |  |
|  | 6 | 1 | 79981634 | 79981634 | 0 | 0 |  |
|  | 9 | 61 | 77276731 | 77807912 | 1 | *VPS13B* |  |
|  | 10 | 105 | 35838530 | 36431208 | 10 | *LATS2, XPO4, EEF1AKMT1, IFT88, CRYL1, GJB6, GJB2, GJA3, ZMYM2, ZMYM5* |  |
|  | 10 | 12 | 42602855 | 42668804 | 0 | 0 |  |
|  | 12 | 79 | 78441984 | 79070188 | 7 | *CSRP1, TNNI1, NAV1, IPO9, SHISA4, RNPEP, ELF3* |  |
|  | 13 | 4 | 49772494 | 49800472 | 0 | 0 |  |
|  | 13 | 82 | 53046392 | 53647951 | 25 | *NPBWR2, OPRL1, RGS19, TCEA2, PRPF6, RF00026, ZNF512B, UCKL1, DNAJC5, ABHD16B, ZBTB46, ZGPAT, ARFRP1, TNFRSF6B, STMN3, GMEB2, FNDC11, SRMS, PTK6, EEF1A2, KCNQ2, CHRNA4, ARFGAP1, BIRC7, YTHDF1* |  |
| SHL | 2 | 67 | 122203171 | 122713621 | 1 | *FSIP2* |  |
|  | 4 | 55 | 68730153 | 69082247 | 20 | *EVX1, RF02043, RF02042, RF02041, RF02040, RF02142, RF02141, RF02140, RF02139, RF02138, RF02137, HOXA10, HOXA6, HOXA3, HOXA2, RF01979, RF01978, RF01976, RF01975, HOXA1* |  |
|  | 6 | 3 | 78164118 | 78171900 | 0 | 0 |  |
|  | 6 | 22 | 78190079 | 78372681 | 1 | *RF00026* |  |
|  | 6 | 5 | 79989614 | 80013968 | 0 | 0 |  |
|  | 6 | 41 | 80045125 | 80283293 | 0 | 0 |  |
|  | 9 | 43 | 77387147 | 77790278 | 1 | *VPS13B* |  |
|  | 10 | 47 | 35839462 | 36132909 | 5 | *LATS2, XPO4, EEF1AKMT1, IFT88, CRYL1* |  |
|  | 10 | 52 | 36173170 | 36431208 | 6 | *CRYL1, GJB6, GJB2, GJA3, ZMYM2, ZMYM5* |  |
|  | 10 | 34 | 42602855 | 42829373 | 0 | 0 |  |
|  | 10 | 29 | 42862671 | 43163671 | 0 | 0 |  |
|  | 12 | 78 | 78449224 | 79070188 | 7 | *CSRP1, TNNI1, NAV1, IPO9, SHISA4, RNPEP, ELF3* |  |
|  | 13 | 72 | 53065617 | 53589429 | 22 | *NPBWR2, OPRL1, RGS19, TCEA2, PRPF6, RF00026, ZNF512B, UCKL1, DNAJC5, ABHD16B, ZBTB46, ZGPAT, ARFRP1, TNFRSF6B, STMN3, GMEB2, FNDC11, SRMS, PTK6, EEF1A2, KCNQ2, CHRNA4* |  |
|  | 20 | 7 | 50349355 | 50424219 | 1 | *GMDS* |  |
| TIB | 2 | 154 | 122210623 | 123369957 | 1 | *FSIP2* |  |
|  | 5 | 75 | 19764108 | 20233040 | 4 | *P4HA2, RF00026, CSF2, IL3* |  |
|  | 10 | 168 | 42182526 | 43525344 | 0 | 0 |  |
|  | 12 | 78 | 78449224 | 79070188 | 7 | *CSRP1, TNNI1, NAV1, IPO9, SHISA4, RNPEP, ELF3* |  |
|  | | 13 | 55 | 53152803 | 53589429 | 18 | *PRPF6, RF00026, ZNF512B, UCKL1, DNAJC5, ABHD16B, ZBTB46, ZGPAT, ARFRP1, TNFRSF6B, STMN3, GMEB2, FNDC11, SRMS, PTK6, EEF1A2, KCNQ2, CHRNA4* |
